# Protective Efficacy Induced by Virus-like Particles Expressing Dense Granule Protein 5 of *Toxoplasma gondii*

**DOI:** 10.3390/vaccines13080787

**Published:** 2025-07-24

**Authors:** Su In Heo, Hae-Ji Kang, Jie Mao, Zhao-Shou Yang, Md Atique Ahmed, Fu-Shi Quan

**Affiliations:** 1 Department of Biomedical Science, Graduate School, Kyung Hee University, Seoul 02447, Republic of Korea; 2 Medical Research Center for Bioreaction to Reactive Oxygen Species and Biomedical Science Institute, Core Research Institute (CRI), Kyung Hee University, Seoul 02447, Republic of Korea; 3 Department of Microbiology, College of Medicine, Dongguk University, Gyeongju 38066, Republic of Korea; 4 The First Affiliated Hospital/The First Clinical Medicine School of Guangdong Pharmaceutical University, Guangdong Pharmaceutical University, Guangzhou 510080, China; 5 ICMR-Regional Medical Research Centre, NER, Dibrugarh 786010, Assam, India; 6 Department of Medical Zoology, School of Medicine, Kyung Hee University, Seoul 02447, Republic of Korea

**Keywords:** *Toxoplasma gondii*, GRA5, virus-like particles, vaccine

## Abstract

**Background:** *Toxoplasma gondii* (*T. gondii*) causes severe disease in immunocompromised individuals and pregnant women, underscoring the urgent need for effective vaccines against toxoplasmosis. The dense granule protein 5 (GRA5) of *T. gondii* plays a key role in parasitic cyst formation. **Methods:** This study evaluated the protective immune responses induced by a virus-like particle (VLP) vaccine expressing the *T. gondii*-derived antigen GRA5 in a mouse model challenged with the ME49 strain of *T. gondii*. GRA5 VLPs were generated using a baculovirus expression system, and VLP formation was confirmed by Western blotting and visualized using transmission electron microscopy. Mice were intranasally immunized with GRA5 VLPs three times at 4-week intervals to induce immune responses, followed by infection with *T. gondii* ME49. **Results:** Intranasal immunization with GRA5 VLPs induced parasite-specific IgG antibody responses in the serum and both IgG and IgA antibody responses in the brain. Compared to the non-immunized group, immunized mice exhibited significantly higher levels of germinal center B cells and antibody-secreting cell responses. Moreover, the VLP vaccine suppressed the production of IFN-γ and IL-6 cytokines, leading to a significant reduction in brain inflammation and decreased cyst counts following lethal challenge with *T. gondii* ME49 infection. **Conclusion:** These findings suggest that the GRA5 VLP vaccine derived from *T. gondii* elicits a protective immune response, highlighting its potential as an effective vaccine candidate against toxoplasmosis.

## 1. Introduction

*Toxoplasma gondii* (*T. gondii*) is an intracellular parasite capable of infecting a wide range of hosts, including humans [1]. Infection in humans is most often initiated by the ingestion of undercooked meat containing viable tissue cysts, exposure to cat feces contaminated with oocysts, or congenital transmission from mother to fetus [2]. It is estimated that approximately one in three people worldwide are infected with *Toxoplasma gondii*, with some areas—like parts of Central Europe—reporting seropositivity rates higher than 60% [3]. In endemic areas, congenital toxoplasmosis remains a significant public health concern, with long-term consequences, including hydrocephalus, chorioretinitis, and neurodevelopmental disorders [4,5]. Current treatment regimens involve combinations of dihydrofolate reductase inhibitors (e.g., pyrimethamine) and sulfonamides, which synergistically inhibit folate metabolism, an essential pathway for *T. gondii* DNA synthesis and replication that target folate metabolism [6]. Despite their efficacy in acute toxoplasmosis, these drugs exhibit several limitations. They are often associated with adverse effects such as bone marrow suppression, hypersensitivity reactions, and hepatotoxicity, necessitating the co-administration of folinic acid to reduce toxicity [7]. Moreover, these treatments are ineffective against bradyzoites residing in tissue cysts, which can persist for life and reactivate under immunosuppressed conditions [8,9,10,11]. Resistance development and poor patient compliance due to prolonged treatment duration further underscore the need for safer and more effective therapeutic strategies.

Given these limitations, the development of a preventive vaccine is crucial to achieve durable protection and reduce the global disease burden of toxoplasmosis. Despite extensive efforts, however, no licensed vaccine is currently available for human use against *T. gondii*. The only commercially available option, Toxovax^®^, is a live-attenuated vaccine used exclusively in sheep to prevent abortion associated with toxoplasmosis [12]. However, Toxovax^®^ has notable limitations, including the potential risk of reversion to virulence, strict cold-chain requirements, limited efficacy across different life stages of the parasite, and an inability to prevent chronic infection [13]. Moreover, due to safety concerns, its use remains restricted to veterinary applications and is unsuitable for humans. These challenges highlight the urgent need to develop a safe, effective, and scalable human vaccine—particularly one capable of targeting the bradyzoite stage and preventing cyst formation and long-term persistence within host tissues.

Recent studies have emphasized the importance of dense granule proteins (GRAs), particularly GRA2 and GRA5, which are secreted into the parasitophorous vacuole and play essential roles in the structural integrity of the cyst wall and in modulating host immune responses [14,15]. Among them, GRA5 has garnered attention as a promising target for chronic toxoplasmosis due to its critical involvement in cyst membrane stabilization and immune evasion. Targeting GRA5 may, therefore, provide a novel approach to disrupt cyst development and enhance long-term protection against latent infection. Despite these implications, the protective efficacy of GRA5 against chronic *T. gondii* infection has not been extensively validated. Most existing studies have focused on acute infection models, where GRA5-based vaccines such as DNA and protein subunit platforms have primarily demonstrated enhanced Th1-type cytokine responses and prolonged survival following lethal challenge [15,16].

However, these approaches often face limitations in terms of immunogenicity, structural stability, and large-scale applicability [17]. In contrast, VLPs offer several distinct advantages. VLPs are non-infectious, self-assembling nanoparticles that closely mimic the size, shape, and repetitive antigen presentation of native viruses, thereby enabling efficient uptake by antigen-presenting cells and robust activation of both B and T cell responses. Additionally, VLPs are highly stable, can elicit strong immune responses without the need for adjuvants, and are amenable to cost-effective and scalable production.

In this study, we applied the VLP platform to express GRA5, marking the first attempt to incorporate this antigen into a VLP-based vaccine. By doing so, we aimed to leverage the immunological and practical advantages of VLPs to enhance vaccine efficacy against chronic toxoplasmosis, particularly by targeting the tissue cyst of the *T. gondii*.

In the present study, we developed a recombinant VLP vaccine expressing the GRA5 antigen and evaluated its protective efficacy against *T. gondii* ME49 in mice. To our knowledge, this represents the first attempt to incorporate GRA5 into a virus-like particle platform. Our findings suggest that the GRA5-containing VLP vaccine may be an effective candidate for the prevention of chronic toxoplasmosis.

## 2. Materials and Methods

### 2.1. Research Ethics Statement

All animal studies were approved by the Kyung Hee University Institutional Animal Care and Use Committee (IACUC, approval number: KHSASP-24-487) and conducted in accordance with institutional guidelines. Mice exhibiting ≥ 20% body weight loss were humanely euthanized via CO_2_ inhalation. Efforts were undertaken to ensure the welfare of the animals and minimize discomfort during the study.

### 2.2. Experimental Animals and Parasite Strains

Female BALB/c mice aged six weeks (purchased from NarabioTech, Seoul, Republic of Korea) were maintained in a pathogen-free facility under regulated light/dark cycles (12 h each) and were provided with unrestricted access to standard diet and water. *T. gondii* ME49 cysts used for both infection and antigen were maintained in the laboratory through BALB/c mice. Briefly, brains from mice previously infected with *T. gondii* ME49 were harvested and homogenized in PBS. A volume of 100 μL of the brain homogenate was intraperitoneally injected into naïve BALB/c mice. Four weeks post-infection, brain tissues were collected, and tissue cysts were purified using a sucrose gradient method [18]. To ensure a consistent and reliable supply of infectious cysts for subsequent experiments, cyst amplification was initiated four weeks prior to the planned challenge infection.

### 2.3. Construction and VLP Production

Total RNA was extracted from *T. gondii* isolated from infected mice using the RNeasy Mini Kit (Qiagen), and complementary DNAs encoding M1 or GRA5 were synthesized as previously described [19]. To generate recombinant baculoviruses (rBVs) expressing M1 or GRA5, the bacmid DNA was introduced into Sf9 insect cells utilizing the Cellfectin II transfection reagent (Invitrogen) [15]. VLPs containing M1 and GRA5 were produced by co-infecting Sf9 cells (2.5 × 10^6^ cells/mL) with M1 rBVs and GRA5 rBVs at a 1:3 ratio and then cultured at 27 °C for 2 days [19]. Culture supernatants were collected by centrifugation, and VLPs were purified by ultracentrifugation through density separation on a sucrose gradient. GRA5 VLPs were suspended in phosphate-buffered saline after purification, stored at 4 °C, and analyzed for protein content using the Bicinchoninic Acid assay [19]. To evaluate the cleanliness of the VLP preparations, Sf9 cells were exposed to either refined or crude VLP samples following established protocols [20]. A separate group of Sf9 cells received no treatment and served as negative control. Over a 7-day observation period, cellular responses to inoculation were tracked, and representative images were captured using a light microscope (Leica Microsystems, Wetzlar, Germany).

### 2.4. VLP Characterization

The formation of VLPs was confirmed through both transmission electron microscopy (TEM) and immunoblotting techniques [21]. For ultrastructural observation, VLP samples were placed on carbon film-coated grids, stained with 2% uranyl acetate, and examined using a JEOL JEM-1000BEF electron microscope operating at 1000 kV (JEOL Ltd., Tokyo, Japan). For protein detection, VLPs were resolved via SDS–PAGE, transferred onto nitrocellulose membranes, and blocked with 5% non-fat milk solution. Membranes were then incubated overnight at 4 °C with either anti-*T. gondii* polyclonal serum (diluted 1:500) or anti-M1 antibody (1:1000). After washing, HRP-linked secondary IgG antibodies (1:2000) were added, and the protein bands were detected using an enhanced chemiluminescence system and visualized with the ChemiDoc MP imaging platform (Bio-Rad, Hercules, CA, USA) [21].

### 2.5. GRA5 VLP Immunization and Challenge Infection in Mice

Mice were randomly divided into three groups, with six animals per group (n = 6). The GRA5 VLP group received three intranasal immunizations (50 µg each) at 4-week intervals. Four weeks after the final immunization, mice in the Naïve + Cha and GRA5 VLP groups were orally challenged with 450 cysts of *T. gondii* ME49. At 27 days after challenge infection, the mice were sacrificed to evaluate vaccine efficacy.

### 2.6. Sample Collection

One week following each immunization, blood was obtained from the retro-orbital sinus. Samples were left to clot at an ambient temperature for 30 min, then centrifuged at 3000 rpm for 10 min to isolate serum, which was subsequently stored at 4 °C. On day 27 after the infection, animals were euthanized, and both brain and spleen tissues were collected. Brain samples were mechanically dissociated in phosphate-buffered saline (PBS) using a syringe and mesh filter, then centrifuged at 2000 rpm for 10 min to remove debris. The supernatant was used for analysis of pro-inflammatory cytokine and antibody responses, whereas the pellet was mixed with a sucrose solution and subjected to ultracentrifugation for brain cyst quantification. Spleen tissues were processed using the same method as for brain tissues to obtain the supernatant. Splenocytes were isolated through red blood cell (RBC) lysis and subsequently used for flow cytometric analysis of immune cell populations and for antibody-secreting cell (ASC) assays. All samples were prepared according to established protocol [21,22,23].

### 2.7. Enzyme-Linked Immunosorbent Assay (ELISA)

Assessment of antibody responses specific to *T. gondii* was conducted according to a previously reported method [24]. Ninety-six-well flat-bottom plates were pre-coated with *T. gondii* ME49 antigen at a concentration of 5 μg/mL and subsequently blocked with 0.2% gelatin to prevent non-specific binding. Primary samples included diluted serum (1:50), brain extracts (1:10), and spleen supernatants (1:5). Following incubation, HRP-labeled anti-mouse IgG antibodies (1:3000 dilution) were applied. Color development was achieved using o-phenylenediamine dihydrochloride as a substrate, and absorbance was quantified at 490 nm using a microplate spectrophotometer (EZread 400, Biochrom, Cambridge, UK).

### 2.8. Antibody-Secreting Cell (ASC) Response

Antibody-secreting cell (ASC) responses were assessed according to a previously established method [25]. Ninety-six-well plates were first coated with lysate antigen *from T. gondii* ME49 (5 μg/mL) and then blocked with 0.2% gelatin to reduce background binding. Splenocytes were plated at a density of 5 × 10^6^ cells per well and incubated for 5 days at 37 °C in a humidified atmosphere containing 5% CO_2_. After thorough washing, horseradish peroxidase (HRP)-linked anti-mouse IgG or IgA antibodies (Southern Biotech, Birmingham, AL, USA) were applied, and the resulting antibody production was quantified using an ELISA-based detection method.

### 2.9. Germinal Center (GC) B Cell Response

Germinal center (GC) B cell responses were evaluated following a modified protocol based on previous reports [25]. Splenocytes obtained from both vaccinated and control groups were stimulated with 5 μg/mL of *T. gondii* ME49 lysate for 2 h at 37 °C to activate antigen-specific GC B cells. After antigen exposure, cells were rinsed with PBS and treated with an Fc receptor blocker to minimize non-specific binding of antibodies. The cells were then labeled using fluorescent monoclonal antibodies targeting CD45 (FITC, cat. #553079) and GL7 (PE, cat. #561530) (BD Biosciences, San Jose, CA, USA), markers commonly used for identifying GC B cell activation. The proportion of GC B cells expressing CD45^+^GL7^+^ was determined using flow cytometric analysis on an Accuri C6 instrument (BD Biosciences, San Jose, CA, USA).

### 2.10. Protective Efficacy

Vaccine-mediated protection by GRA5 VLPs was assessed via oral challenge with a lethal dose of *T. gondii* ME49 suspended in PBS. Body weight and survival rate were monitored at consistent time points every 3 days until 27 days post-infection. At the endpoint, from the resulting suspension containing purified brain cysts, a 10 μL aliquot was placed on a microscope slide and examined under a light microscope. Cyst counts were performed in triplicate per sample, and the average number of cysts per mouse was calculated and compared across groups. Furthermore, levels of pro-inflammatory cytokines, including IFN-γ and IL-6, were quantified in brain homogenate supernatants via ELISA. Absorbance readings were taken at 562 nm using a microplate spectrophotometer.

### 2.11. Statistical Analysis

Statistical evaluations were conducted using GraphPad Prism software (version 10.1.2, USA). Comparisons between groups utilized unpaired two-sided *t*-tests, one-way or two-way analysis of variance (ANOVA), followed by suitable post-hoc analyses as needed. Results are expressed as mean values ± standard deviation (SD). Differences were considered statistically significant at thresholds of * *p* < 0.05, ** *p* < 0.01, and ** *p* < 0.001.

## 3. Results

### 3.1. GRA5 Construct and VLP Generation

**Figure 1:**
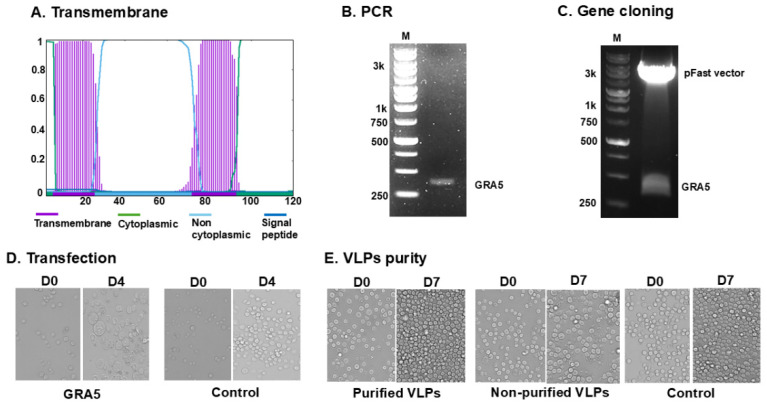
Construction of GRA5 and generation of recombinant baculovirus. The transmembrane topology of GRA5 was predicted using Phobius (**A**). PCR amplification yielded the expected GRA5 band (363 bp) (**B**). The gene was inserted into the pFastBac vector and verified through restriction enzyme digestion (**C**). Sf9 cells transfected with the GRA5 bacmid showed morphological changes at 4 days post-transfection compared to controls (**D**). VLP purity was confirmed by infecting Sf9 cells with purified or non-purified VLPs, including controls where no VLP was added. The Sf9 cells were observed at day 7 post-reaction with VLPs to assess Sf9 cell infectivity (**E**).

**Figure 2:**
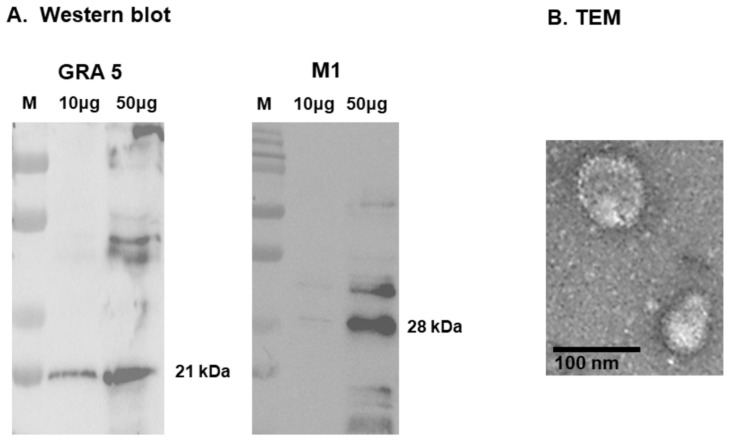
Characterization of GRA5 VLPs. Western blot analysis confirmed the expression of M1 (28 kDa) and GRA5 (21 kDa) antigens in the VLPs (**A**). TEM verified the morphological structure of GRA5 VLPs (**B**).

Phobius software was used to predict the transmembrane domains and signal peptides of GRA5, providing essential information for proper antigen expression and surface localization in the VLP (Figure 1A). PCR amplification resulted in a 363 bp full-length GRA5 fragment (Figure 1B), which was cloned into the pFastBac vector. The construct was verified by double digestion with BamHI and XbaI, producing expected restriction patterns (Figure 1C), confirming successful insertion of the target gene. Bacmid DNA was generated and transfected into *Spodoptera frugiperda* (Sf9) cells, which exhibited morphological changes on day 4 post-infection between groups (Figure 1D). Sf9 cells transfected with the recombinant GRA5 gene exhibited a more than twofold increase in cell size compared to day 0, indicating successful viral entry and antigen expression. The infected cells appeared swollen and were morphologically distinct. In contrast, control cells showed no signs of baculovirus-induced changes and continued to proliferate without evidence of cytopathic effects. Recombinant baculoviruses were harvested and co-infected with influenza M1 baculovirus into Sf9 cells. The purified VLPs and control showed significant proliferation of SF9 cells on day 7 post-inoculation with VLPs, indicating that the baculoviruses had been removed (Figure 1E). However, non-purified VLPs exhibited cell death and did not proliferate, indicating the presence of baculovirus (Figure 1E). Western blot analysis confirmed the presence of GRA5 (21 kDa) and M1 (28 kDa) in the VLPs (Figure 2A), and TEM verified VLP morphology, confirming successful VLP formation (Figure 2B).

### 3.2. Antigen Expression and Structural Validation of GRA5 VLPs

Western blot analysis confirmed the successful expression of both GRA5 and M1 proteins in the VLPs, with distinct bands detected at the expected molecular weights of 21 kDa for GRA5 and 28 kDa for the influenza matrix protein M1 (Figure 2A). To further assess the structural integrity and morphology of the VLPs, TEM was performed. As shown in Figure 2B, spherical particles with a size range of 100–200 nm, characteristic of virus-like particles, were observed. Surface-localized GRA5 antigen expression was also evident, confirming successful antigen display.

### 3.3. T. gondii-Specific IgG Antibody Responses in Serum and Brain

**Figure 3:**
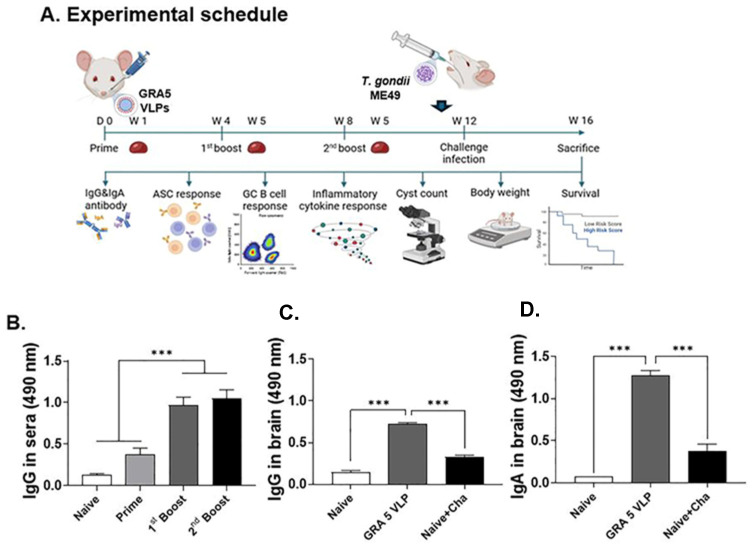
Experimental schedule and antibody responses in sera and brain. GRA5 VLPs were administered to mice via the intranasal route on three occasions spaced four weeks apart, with blood samples obtained one week following each vaccination (**A**). ELISA revealed significantly increased *T. gondii*-specific IgG in sera (**B**), with elevated IgG (**C**) and IgA (**D**) also detected in brain homogenate supernatants. Results are expressed as mean ± SD; significance indicated by *** *p* < 0.001.

The mice were administered three intranasal vaccinations of GRA5 VLPs at intervals of four weeks and subsequently challenged with *T. gondii* ME49 at the twelfth week. Serum samples were collected one week after each immunization to monitor antibody production. At week 16, which is four weeks after infection challenge, the animals were euthanized to analyze immune responses and assess protection (Figure 3A). The vaccinated group displayed significantly increased levels of *T. gondii*-specific IgG antibodies in both serum (Figure 3B) and brain tissue (Figure 3C) compared to the Naïve + Challenge group. In addition, mucosal IgA levels in the brain were elevated in the GRA5 VLP group (Figure 3D). These results collectively indicate that intranasal immunization with GRA5 VLPs effectively stimulates antibody responses, which may play a crucial role in limiting *T. gondii* dissemination and persistence.

### 3.4. T. gondii-Specific IgG and IgA Responses in Spleen Tissue and ASC

**Figure 4:**
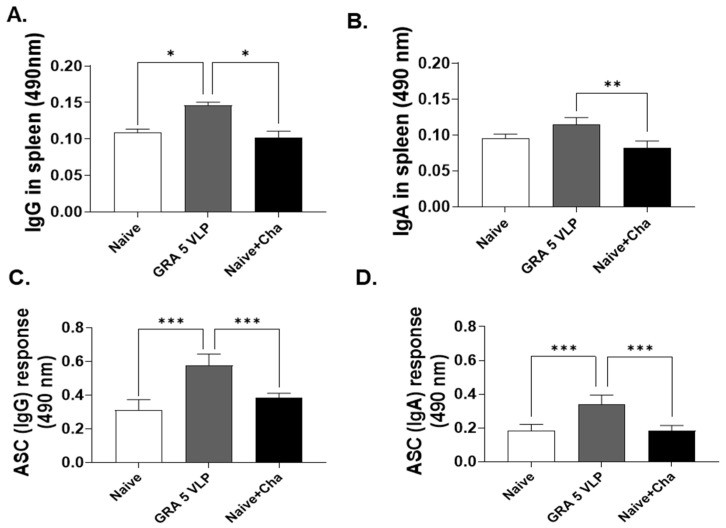
Antibody and ASC responses in the spleen. Levels of *T. gondii*-specific IgG (**A**) and IgA (**B**) antibodies in spleen samples were quantified using ELISA. Additionally, splenocytes activated with *T. gondii* lysate antigen were evaluated for IgG (**C**) and IgA (**D**) secretion through an antibody-secreting cell (ASC) assay. Results are expressed as mean ± SD; * *p* < 0.05, ** *p* < 0.01, and *** *p* < 0.001.

Spleen homogenates were prepared to assess spleen-derived IgG and IgA antibody responses, and splenocytes were collected and cultured to evaluate IgG- and IgA-secreting ASC responses. As shown in Figure 4, GRA5 VLP-immunized mice exhibited significantly elevated levels of *T. gondii*-specific IgG (Figure 4A) and IgA (Figure 4B) within spleen tissue compared to the Naïve + Cha group. Additionally, the functional capacity of B cells was assessed by quantifying IgG- and IgA-secreting ASCs using ELISA. The GRA5 VLP group showed a marked increase in both IgG- (Figure 4C) and IgA-secreting (Figure 4D) ASCs compared to controls. This indicates that GRA5 VLP immunization not only enhanced antibody titers in spleen tissue but also promoted the differentiation of B cells into active plasma cells.

### 3.5. T. gondii-Specific ASC (IgG, IgA) Responses

**Figure 5:**
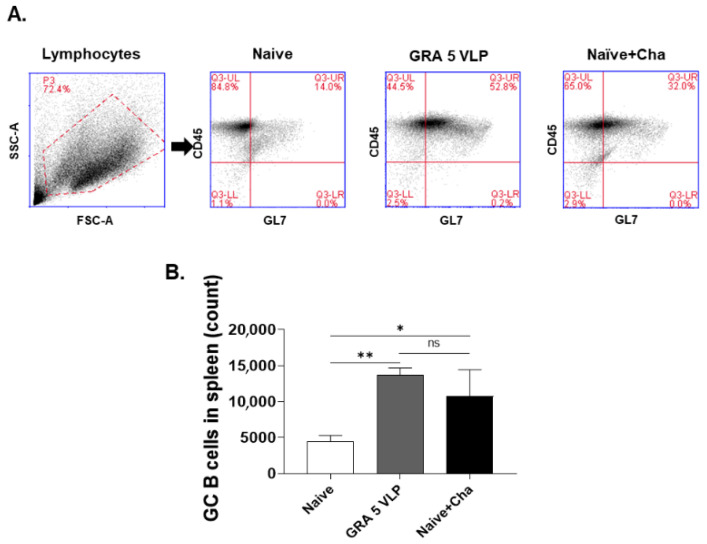
Evaluation of germinal center (GC) B cell subsets within the spleen. Splenocytes harvested from vaccinated mice were exposed to *T. gondii* lysate antigen, labeled with specific markers for GC B cells (CD45^+^/GL7^+^), and subjected to analysis. Representative flow cytometry data (**A**) and the quantified proportions of GC B cells in the spleen (**B**) are presented. Results are expressed as mean ± SD; * *p* < 0.05, ** *p* < 0.01.

GC B cells were identified using flow cytometry based on GL7^+^CD45^+^ gating, and the frequency was markedly elevated in the immunized group, indicating active GC formation following immunization (Figure 5A). A significant increase in germinal center (GC) B cell populations was observed in the spleens of GRA5 VLP-immunized mice compared to the Naïve + Cha group (Figure 5B).

### 3.6. Protective Efficacy of GRA5 VLPs Against T. gondii ME49 Infection

**Figure 6:**
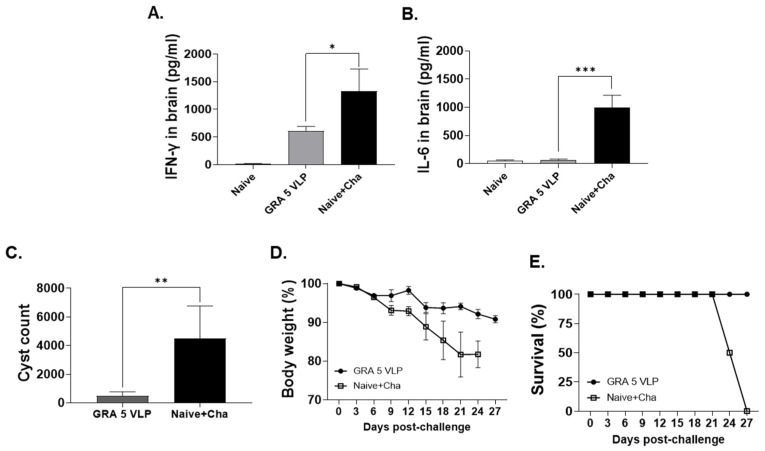
Protective effect of GRA5 VLP immunization against *T. gondii* ME49 infection. Brain homogenate supernatants were analyzed for IFN-γ (**A**) and IL-6 (**B**). Following challenge infection, brain cyst burden (**C**), body weight changes (**D**), and survival rates (**E**) were evaluated. Results are expressed as mean ± SD; * *p* < 0.05, ** *p* < 0.01, and *** *p* < 0.001.

Immunization with GRA5 VLPs led to a substantial reduction in brain levels of pro-inflammatory cytokines. Specifically, IFN-γ levels were reduced from 1328 pg/mL in the Naïve + Cha group to 604 pg/mL in the GRA5 VLP group—representing a 2.2-fold decrease (Figure 6A). Similarly, IL-6 levels decreased from 997 pg/mL to 66 pg/mL, showing a dramatic 15-fold reduction (Figure 6B). These reductions suggest that GRA5 VLP vaccination effectively dampens the inflammatory response in the brain following *T. gondii* infection. Additionally, GRA5 VLP immunization markedly reduced the number of brain cysts after *T. gondii* ME49 infection. While the Naïve + Cha group exhibited an average of 4500 brain cysts, only 483 cysts were detected in the GRA5 VLP group, corresponding to an approximate 9.3-fold reduction (Figure 6C). This substantial decrease demonstrates the vaccine’s efficacy in preventing chronic tissue cyst formation in the brain. Ultimately, GRA5 VLP-immunized mice exhibited 100% survival throughout the 35-day observation period, with only a modest 9% reduction in body weight following *T. gondii* ME49 challenge (Figure 6D,E). In contrast, mice in the Naïve + Cha group showed a 20.8% decrease in body weight and all succumbed to infection by day 27 post-challenge.

## 4. Discussion

*T. gondii* infects billions of people worldwide [1], posing significant risks to immunocompromised individuals and pregnant women. The drawbacks of existing toxoplasmosis therapies, including side effects and the emergence of drug-resistant strains [8], underscore the critical necessity for creating effective vaccination strategies. In the present study, we developed VLPs containing the *T. gondii* GRA5 antigen, aiming to harness the immunogenic potential of VLPs to induce protective immunity against chronic toxoplasmosis. Vaccination using GRA5 VLPs conferred effective protection against *T. gondii* ME49 infection, leading to a notable decrease in cyst numbers and 100% survival rate.

Previous studies from our laboratory focused on invasion-associated antigens, such as MIC8, ROP, and IMC, for the generation of VLPs, resulting in protective outcomes [26]. In contrast, the present study explored GRA5, a dense GRA involved in cyst stabilization, as a novel antigenic target to enhance protection against *T. gondii* ME49. Unlike invasion-stage antigens, which are transiently exposed, GRA5 is continuously and stably expressed throughout the chronic phase of infection and is a key component of the cyst wall.

Based on this rationale, immunization with GRA5-expressing VLPs provided immune stimulation, leading to the sustained induction of GC B cell responses and generation of *T. gondii*-specific antibodies. This observation is consistent with our previous findings using CST1, another chronic-stage cyst wall protein, which resulted in significantly elevated GC B cell responses compared to those of VLPs expressing acute-phase antigens such as ROP18 or MIC8 [27,28]. These results support the notion that cyst-associated antigens such as GRA5 and CST1 are effective in promoting durable immunity against *T. gondii* ME49.

Although antigen doses of 100 μg are commonly used in *T. gondii* VLP vaccine studies [18], we intentionally selected a lower dose of 50 μg to evaluate the inherent immunogenicity of GRA5. To enhance immune activation despite the reduced dose, we used the intranasal route, which is known to effectively induce both mucosal and systemic immune responses, even at lower doses. Mucosal immunity is important in *T. gondii* ME49 infection, which is acquired through mucosal surfaces and leads to cyst formation in the brain. In the present study, intranasal immunization with GRA5 VLPs successfully induced mucosal IgA responses and systemic immunity, significantly reducing the number of brain cysts and demonstrating protective efficacy against *T. gondii* ME49.

Another major challenge in *T. gondii* ME49 infection is the rapid escalation of pro-inflammatory cytokines in the brain, which can ultimately cause severe tissue damage [29]. Therefore, an ideal vaccine should not only elicit protective immunity but also minimize excessive inflammation to avoid collateral tissue damage [30]. Our findings show that immunization with GRA5 VLPs significantly suppressed pro-inflammatory cytokine responses, especially IFN-γ and IL-6. This reduction is presumed to result from a decreased cyst count in the brain, as a lower number of *T. gondii* likely triggers a weaker pro-inflammatory response, leading to an overall decrease in cytokine levels. These findings suggest that the immune responses induced by GRA5 VLP immunization demonstrated through the aforementioned results effectively contributed to suppressing *T. gondii* activity and controlling replication.

Although the results are promising, several limitations of this study should be considered and explored in future studies. First, all of the experiments were conducted in mice, which may not fully recapitulate the complexity of human immune responses. Second, considering the extensive antigenic diversity of *T. gondii*, the use of a single antigen, such as GRA5, may not provide broad or sufficient protection in humans. To facilitate clinical applications, further research is warranted to investigate combinations of multiple GRA antigens or other stage-specific proteins to enhance vaccine efficacy. Third, although this study focused on GC B cell responses as a key indicator of humoral immunity, other important immune parameters, such as T cell-mediated responses, were not evaluated. A comprehensive analysis of cellular immunity involving CD4 + and CD8 + T cells is necessary to fully characterize the protective mechanisms induced by GRA5 VLPs.

## 5. Conclusions

GRA5 VLP immunization induced the expansion of GC B cells in the spleen, leading to protective immunity through the induction of *T. gondii*-specific IgG and IgA antibodies and the suppression of pro-inflammatory cytokines in the brain. These immune responses resulted in an 82% reduction in the brain cyst count and 100% survival, demonstrating the potential of GRA5 VLPs as a promising vaccine candidate for chronic toxoplasmosis.

## Data Availability

Data will be made available on request.

## References

[1] Tenter A.M., Heckeroth A.R., Weiss L.M. (2000). *Toxoplasma gondii*: From animals to humans. Int. J. Parasitol..

[2] Shapiro K., Bahia-Oliveira L., Dixon B., Dumètre A., de Wit L.A., VanWormer E., Villena I. (2019). Environmental transmission of *Toxoplasma gondii*: Oocysts in water, soil and food. Food Waterborne Parasitol..

[3] Kota A.S., Shabbir N. (2023). Congenital toxoplasmosis. StatPearls [Internet].

[4] Diebler C., Dusser A., Dulac O. (1985). Congenital toxoplasmosis: Clinical and neuroradiological evaluation of the cerebral lesions. Neuroradiology.

[5] Hutson S.L., Wheeler K.M., McLone D., Frim D., Penn R., Swisher C.N., Heydemann P.T., Boyer K.M., Noble A.G., Rabiah P. (2015). Patterns of hydrocephalus caused by congenital *Toxoplasma gondii* infection associate with parasite genetics. Clin. Infect. Dis..

[6] Hopper A.T., Brockman A., Wise A., Gould J., Barks J., Radke J.B., Sibley L.D., Zou Y., Thomas S. (2019). Discovery of selective *Toxoplasma gondii* dihydrofolate reductase inhibitors for the treatment of toxoplasmosis. J. Med. Chem..

[7] Ben-Harari R.R., Goodwin E., Casoy J. (2017). Adverse event profile of pyrimethamine-based therapy in toxoplasmosis: A systematic review. Drugs RD.

[8] Dunay I.R., Gajurel K., Dhakal R., Liesenfeld O., Montoya J.G. (2018). Treatment of toxoplasmosis: Historical perspective, animal models, and current clinical practice. Clin. Microbiol. Rev..

[9] Aspinall T.V., Joynson D.H., Guy E., Hyde J.E., Sims P.F. (2002). The molecular basis of sulfonamide resistance in *Toxoplasma gondii* and implications for the clinical management of toxoplasmosis. J. Infect. Dis..

[10] Alday P.H., Doggett J.S. (2017). Drugs in development for toxoplasmosis: Advances, challenges, and current status. Drug Des. Dev. Ther..

[11] Murata Y., Sugi T., Weiss L.M., Kato K. (2017). Identification of compounds that suppress *Toxoplasma gondii* tachyzoites and bradyzoites. PLoS ONE.

[12] Innes E.A., Bartley P.M., Maley S., Katzer F., Buxton D. (2009). Veterinary vaccines against *Toxoplasma gondii*. Memórias Do Inst. Oswaldo Cruz.

[13] Chu K.-B., Quan F.-S. (2021). Advances in *Toxoplasma gondii* vaccines: Current strategies and challenges for vaccine development. Vaccines.

[14] Fox B.A., Guevara R.B., Rommereim L.M., Falla A., Bellini V., Pètre G., Rak C., Cantillana V., Dubremetz J.-F., Cesbron-Delauw M.-F. (2019). *Toxoplasma gondii* parasitophorous vacuole membrane-associated dense granule proteins orchestrate chronic infection and GRA12 underpins resistance to host gamma interferon. MBio.

[15] Ching X.T., Fong M.Y., Lau Y.L. (2016). Evaluation of immunoprotection conferred by the subunit vaccines of GRA2 and GRA5 against acute toxoplasmosis in BALB/c mice. Front. Microbiol..

[16] Ching X.T., Fong M.Y., Lau Y.L. (2017). Evaluation of the protective effect of deoxyribonucleic acid vaccines encoding granule antigen 2 and 5 against acute Toxoplasmosis in BALB/c Mice. Am. J. Trop. Med. Hyg..

[17] Eom G.-D., Chu K.-B., Kang H.-J., Kim M.-J., Yoon K.-W., Mao J., Lee S.-H., Ahmed M.A., Moon E.-K., Quan F.-S. (2023). Protective mucosal and systemic immunity induced by virus-like particles expressing *Toxoplasma gondii* cyst wall protein. PLoS ONE.

[18] Kang H.J., Chu K.B., Lee S.H., Kim M.J., Park H., Jin H., Moon E.K., Quan F.S. (2020). *Toxoplasma gondii* virus-like particle vaccination alleviates inflammatory response in the brain upon T gondii infection. Parasite Immunol..

[19] Lee S.-H., Chu K.-B., Kang H.-J., Quan F.-S. (2019). Virus-like particles containing multiple antigenic proteins of *Toxoplasma gondii* induce memory T cell and B cell responses. PLoS ONE.

[20] Chu K.-B., Kang H.-J., Yoon K.-W., Lee H.-A., Moon E.-K., Han B.-K., Quan F.-S. (2021). Influenza virus-like particle (VLP) vaccines expressing the SARS-CoV-2 S glycoprotein, S1, or S2 domains. Vaccines.

[21] Lee S.-H., Kim A.-R., Lee D.-H., Rubino I., Choi H.-J., Quan F.-S. (2017). Protection induced by virus-like particles containing *Toxoplasma gondii* microneme protein 8 against highly virulent RH strain of Toxoplasma gondii infection. PLoS ONE.

[22] Kang H.-J., Lee S.-H., Kim M.-J., Chu K.-B., Lee D.-H., Chopra M., Choi H.-J., Park H., Jin H., Quan F.-S. (2019). Influenza virus-like particles presenting both *Toxoplasma gondii* ROP4 and ROP13 Enhance Protection against *T. gondii* infection. Pharmaceutics.

[23] Kang H.-J., Mao J., Kim M.-J., Yoon K.-W., Eom G.-D., Chu K.-B., Moon E.-K., Quan F.-S. (2023). The detection of *Toxoplasma gondii* ME49 infections in BALB/c mice using various techniques. Parasites Hosts Dis..

[24] Kim M.-J., Chu K.B., Yoon K.-W., Kang H.-J., Lee D.-H., Moon E.-K., Quan F.-S. (2024). Virus-like particles expressing microneme-associated antigen of *Plasmodium berghei* confer better protection than those expressing apical membrane antigen 1. Parasites Hosts Dis..

[25] Yoon K.-W., Chu K.-B., Kang H.-J., Kim M.-J., Eom G.-D., Quan F.-S. (2022). Orally administrated recombinant vaccinia virus displaying ROP4 induces protection against *Toxoplasma gondii* challenge infection. Vaccines.

[26] Lee S.-H., Kang H.-J., Lee D.-H., Quan F.-S. (2019). Protective immunity induced by incorporating multiple antigenic proteins of *Toxoplasma gondii* into influenza virus-like particles. Front. Immunol..

[27] Eom G.-D., Chu K.B., Mao J., Yoon K.-W., Kang H.-J., Moon E.-K., Kim S.S., Quan F.-S. (2024). Heterologous immunization targeting the CST1 antigen confers better protection than ROP18 in mice. Nanomedicine.

[28] Mao J., Eom G.-D., Yoon K.-W., Heo S.I., Kang H.-J., Chu K.B., Moon E.-K., Quan F.-S. (2025). Protective humoral immunity induced by virus-like particles expressing *Toxoplasma gondii* CST1 or MIC8. Acta Trop..

[29] Chen L., Deng H., Cui H., Fang J., Zuo Z., Deng J., Li Y., Wang X., Zhao L. (2017). Inflammatory responses and inflammation-associated diseases in organs. Oncotarget.

[30] Yoon C., Ham Y.S., Gil W.J., Yang C.-S. (2024). Exploring the potential of *Toxoplasma gondii* in drug development and as a delivery system. Exp. Mol. Med..

